# CFTR Modulates Hypothalamic Neuron Excitability to Maintain Female Cycle

**DOI:** 10.3390/ijms241612572

**Published:** 2023-08-08

**Authors:** Yong Wu, Yanting Que, Junjiang Chen, Lei Sun, Jinghui Guo, Ye Chun Ruan

**Affiliations:** 1 Department of Biomedical Engineering, The Hong Kong Polytechnic University, Hong Kong SAR, Chinaguojinghui@cuhk.edu.cn (J.G.); 2 Department of Physiology, Jinan University, Guangzhou 510632, China; 3 School of Medicine, The Chinese University of Hong Kong, Shenzhen 518172, China

**Keywords:** hypothalamus, excitability, CFTR, Cl^−^

## Abstract

Cystic fibrosis transmembrane conductance regulator (CFTR), known as an epithelial Cl^−^ channel, is increasingly noted to be expressed in the nervous system, although whether and how it plays a role in neuronal excitability is unclear. Given the association of CFTR with fertility, we tested here possible involvement of CFTR in regulating hypothalamic neuron excitability. Patch-clamp and Ca^2+^ imaging showed that pharmacological inhibition of CFTR evoked electrical pulses and Ca^2+^ spikes in primary rat hypothalamic neurons, which was dependent on extracellular Cl^−^. Hypothalamic neurons in brain-slice preparations from adult female mice with CFTR mutation (DF508) exhibited significantly reduced electrical pulses as compared to the wild-type controls. Removal of extracellular Cl^−^ eliminated hypothalamic electrical pulses in the wild-type brain slices, which was reversible by subsequent addition of Cl^−^. In adult female mice, Ca^2+^ indicator (GCaMP6s)-based fiber-photometry showed that hypothalamic Ca^2+^ activities in vivo were enhanced at the proestrus/estrus phase as compared to the diestrus phase of the female cycle. Such estrus-associated hypothalamic activities were largely diminished in DF508 female mice, together with delayed puberty and disturbed female cycles. Therefore, these findings suggest a critical role of CFTR in modulating hypothalamic neuron excitability, which may account for the disturbed female cycles and reduced female fertility associated with CFTR mutations.

## 1. Introduction

Cystic fibrosis transmembrane conductance regulator (CFTR), an anion channel encoded by a single gene located in chromosome seven in humans, was originally identified as an epithelial ion channel responsible for Cl^−^ secretion driving water movement across the epithelium [1,2,3]. Defects in CFTR, as seen in cystic fibrosis (CF), a genetic disease caused by mutations of CFTR [4], lead to disturbance in epithelial homeostasis and thus complications of epithelium-enriched organs, such as airway obstruction/inflammation, gastrointestinal problems, and reproductive failures [5,6]. Notably, increasing evidence has shown CFTR expression in non-epithelial tissues as well. Systemic clinical manifestations are documented in CF, including insulin insufficiency/diabetes [7,8] and osteopenia/osteoporosis [9,10], many of which are not directly related to epithelial functions. Previously, we demonstrated that CFTR mediates Cl^−^ efflux in pancreatic islet β cells, which directly contributes to glucose-stimulated action potentials and thus β cell excitability [11]. 

The hypothalamus–pituitary–gonad axis is a key regulatory system for gonad development and reproductive functions. Gonadotropin-releasing hormone (GnRH) is released from GnRH neurons in the hypothalamus to stimulate the production of follicle-stimulating hormone and luteinizing hormone from the pituitary. In females, GnRH pulsatile secretion correlates with GnRH neuron activities to regulate female hormonal homeostasis maintaining the menstrual/female cycle [12]. Of note, CFTR expression has been reported in the hypothalamus [13,14,15,16,17]. Puberty delay, disturbed female cycle, and reproductive problems are commonly reported in CF and animal models with CFTR deficiency [18,19,20,21]. Given the previous discovery of CFTR in contribution to the excitability of pancreatic islet β cells, we asked whether CFTR could possibly play a role in the excitability of hypothalamic neurons to regulate reproductive endocrine homeostasis. In the present study, using patch-clamp and Ca^2+^ imaging in primary rat hypothalamic neurons and a DF508 mutation-caused CFTR deficient mouse model in conjunction with brain slice patch-clampand Ca^2+^ indicator (GCaMP6s)-based fiber-photometry, we demonstrate here that either CFTR or Cl^−^ environment is essential to hypothalamic electrical pulses and excitability; CFTR deficiency impairs estrus-related hypothalamic neuronal activities in mice.

## 2. Results

### 2.1. CFTR-Inhibition Evokes Electrical Pulses in Primary Rat Hypothalamic Neurons In Vitro

**Figure 1:**
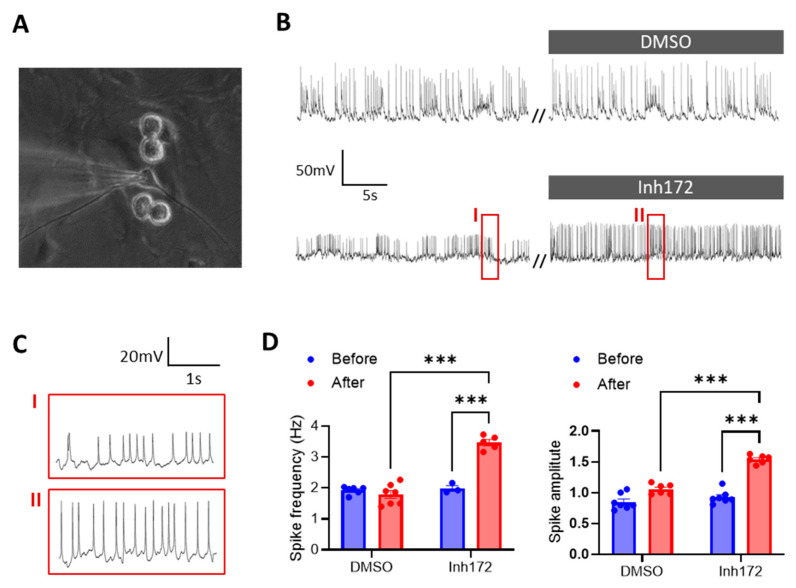
CFTR inhibition evokes electrical pulses in primary rat hypothalamic neurons. (**A**) Brightfield image of primarily cultured rat hypothalamic neurons under patch-clamp. (**B**,**C**) Current-clamp whole-cell recording of membrane potential changes in rat hypothalamic neurons before and after treatment with a selective inhibitor of CFTR, Inh172 (10 μM), or DMSO as the vehicle control. Squared periods (I, II) in (**B**) are enlarged in (**C**). // indicates a break in recording. (**D**) Quantification of the frequency and amplitude of electrical pulses/spikes under different conditions. n = 5–6. *** p < 0.001. Two-tail unpaired Student’s *t*-tests. Data are mean ± s.e.m.

We first used primary cultures of rat hypothalamic neurons (Figure 1A) to test the involvement of CFTR in regulating hypothalamus excitability. In the current-clamp mode of patch-clamp, with no current injected (clamped at 0), the primary hypothalamic neurons exhibited spontaneous electrical pluses/spikes with an averaged amplitude of 30.0 ± 10.1 mV and frequency of 1.9 ± 0.1 Hz. We then added a selective inhibitor of CFTR, Inh172 (10 µM), into the bath solution for the cultured hypothalamic neurons, which shortly caused significant increases in both the amplitude and frequency of the electrical spikes in the cells (Figure 1B–D). DMSO, the vehicle control of the drug, did not change the electrical spikes much in the neurons (Figure 1B,D). It therefore suggested a role of CFTR in modulating the electrical pulses/action potential firing in the hypothalamic neurons.

### 2.2. CFTR-Inhibition Induces Cl^−^-Dependent Ca^2+^ Responses in Primary Rat Hypothalamic Neurons and Glia Cells In Vitro

**Figure 2:**
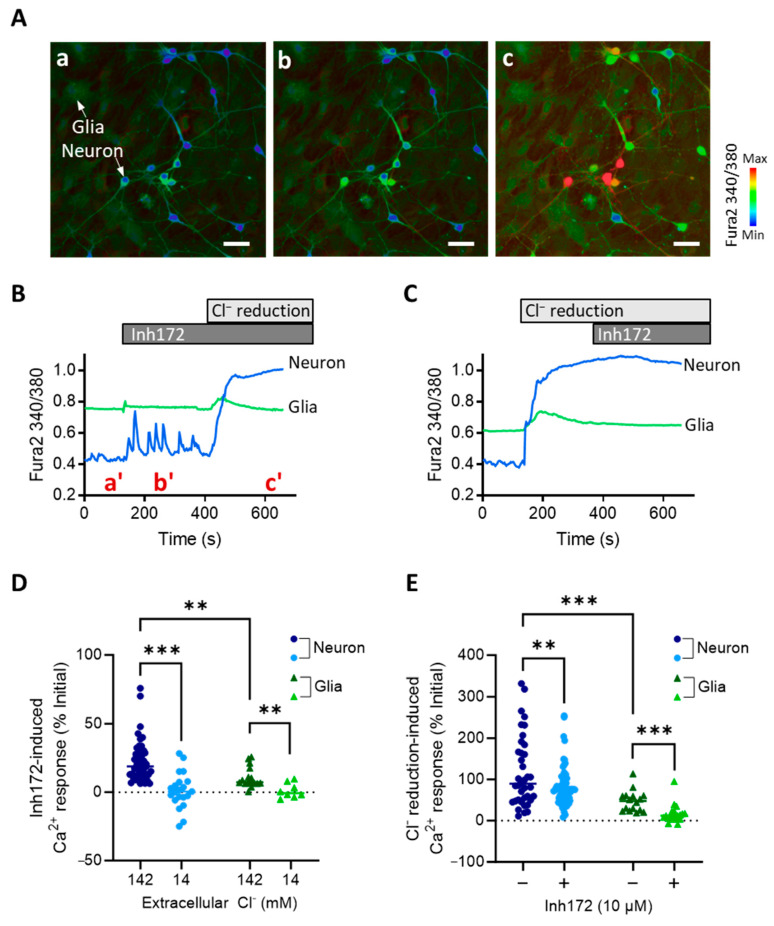
CFTR-inhibition induces Cl^−^-dependent Ca^2+^ responses in primary rat hypothalamic neurons and glia cells (**A**,**B**) Representative fluorescence images (**A**) and time-course traces (**B**) of intracellular Ca^2+^ measurement by Fura-2 in primary rat hypothalamic neuron and glia cells before and after the addition of Inh172 (10 μM) into the bath and subsequently reduction of Cl^−^ from the bath from 142 to 14 mM by perfusion. Images (**a**–**c**) in (**A**) are corresponding to a’, b’, and c’ timepoints indicated in (**B**). (**C**) Time-course traces of Ca^2+^ measurement in the cells treated with Cl^−^ reduction before Inh172. (**D**,**E**) Quantification of Inh172-induced (**D**) and Cl^−^-reduction-induced Ca^2+^ responses under different conditions in the hypothalamic neuron and glia cells. Scale bars, 20 µm. ** p < 0.01, *** p < 0.001. Two-way ANOVA with post hoc tests.

We next performed intracellular Ca^2+^ imaging in the primary hypothalamic neurons to understand how CFTR plays such a role in modulating hypothalamus excitability. Consistent with the above patch-clamp results, the Inh172 (10 µM) treatment of the hypothalamic neurons also elicited Ca^2+^ spikes in the cells (Figure 2A,B). Since CFTR is known as a Cl^−^ channel, such an effect of CFTR inhibition in promoting Ca^2+^ responses might be due to the blockage of Cl^−^ influx. We therefore treated the cells with a protocol to reduce extracellular Cl^−^ from 142 mM by 10 times to 14 mM. Such a Cl^−^ reduction caused a robust and sustained Ca^2+^ elevation in the primary hypothalamic neurons (Figure 2C), suggesting constant Cl^−^ influx into cells suppressing electrical activities with the extracellular Cl^−^ concentration at 142 mM. Notably, after the Ca^2+^ level was elevated by the Cl^−^ reduction treatment, Inh172 (10 µM) no longer induced Ca^2+^ spikes (Figure 2C). Instead, in some cells, the Ca^2+^ level was even lowered by Inh172 (Figure 2C). Comparing the two conditions of 142 and 14 mM extracellular Cl^−^ showed that the Cl^−^-reduction significantly reversed Inh172-induced Ca^2+^ responses in the neurons (Figure 2D). On the other hand, Cl^−^-reduction-evoked Ca^2+^ elevation was partially inhibited when Inh172 was present in the bath (Figure 2E). These results therefore suggest CFTR is a significant part of Cl^−^ conductance, either influx or efflux across the plasma membrane depending on the direction of the Cl^−^ gradient across the membrane. Interestingly, since the primary cultures inevitably contained glia cells, which could be easily differentiated from the neurons based on their morphology in Ca^2+^ imaging (Figure 2A), we analyzed their Ca^2+^ responses too. Similar Ca^2+^ responses to either Inh172 or Cl^−^ reduction were observed in glia cells, although the responses were found to be substantially smaller as compared to those of neuron cells (Figure 2B–E).

### 2.3. CFTR Deficiency or Cl^−^ Removal Impairs Hypothalamic Electrical Firing in Mouse Brain Slices Ex Vivo

**Figure 3:**
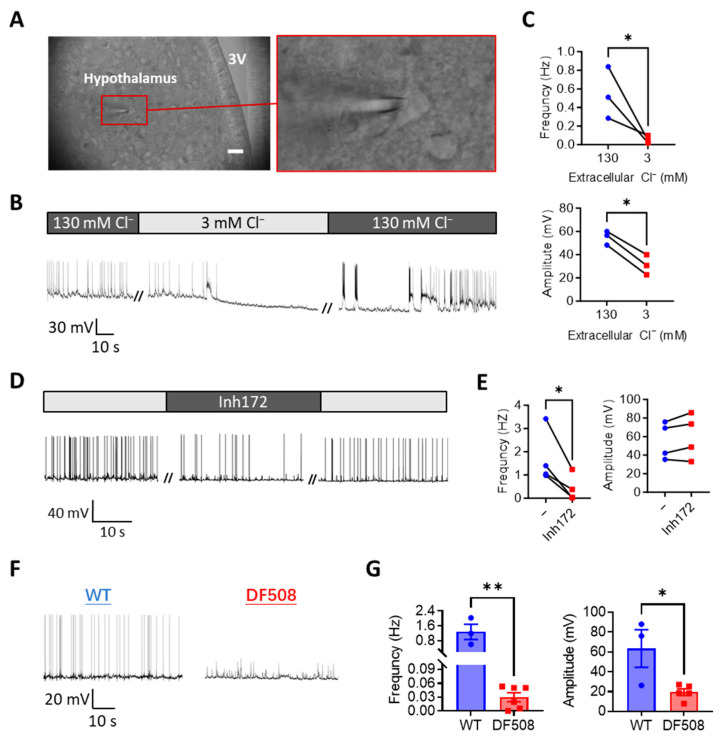
CFTR deficiency or Cl^−^ removal impairs hypothalamic electrical firing in mouse brain slices ex vivo (**A**) Brightfield images of brain-slices from adult female mice with a hypothalamic neuron under patch-clamp. Hypothalamic regions were identified close to the third ventricle (3V), Scale bar, 20 µm. (**B**–**E**) Representative (**B**,**D**) and quantification (**C**,**E**) of current-clamp (at 0 pA) whole-cell recording of membrane potential changes in hypothalamic neurons in brain-slices from wild-type female mice perfused with solutions containing 130 or 3 mM Cl^−^ (**B**,**C**) or Inh172 (10 µM, (**D**,**E**)). // indicates a break of recording. (**F**,**G**) Representative and quantification of current-clamp (at 0 pA) recording of membrane potential changes in hypothalamic neurons in brain-slices from wild-type (WT) and DF508 (CFTR mutant) mice. * p < 0.05, ** p < 0.01. n = 3–5. Two-tail unpaired Student’s *t*-tests. Data are shown as mean ± s.e.m.

We next performed mouse brain slice patch-clamps to further understand the role of CFTR in the hypothalamus. Visible cells within the hypothalamic region in the brain slice (Figure 3A) were chosen for patch-clamp while the preparation was perfused with a physiological solution (see methods). As seen similarly in isolated neurons, under current-clamp at 0 pA, spontaneous electrical spikes from hypothalamic neurons were observed in the brain slice (Figure 3B). We next used a low-Cl^−^ solution to perfuse the preparation. Different from the isolated hypothalamic neurons, the low-Cl^−^ solution treatment resulted in gradual disappearance of the electrical spikes in about 5 min in the brain slices (Figure 3B,C). Importantly, when the perfusion was subsequently switched back to the normal-Cl^−^ solution, the electrical spikes recurred and fully recovered in about 10 min (Figure 3B), suggesting Cl^−^ as a key environmental factor required to generate hypothalamic electrical spikes. Similarly, perfusing Inh172 (10 µM) inhibited the electrical spikes in the brain slices, which were recovered after washing out Inh172 (Figure 3D,E). We then took brain slices from a CFTR mutant mouse model, DF508, and compared their hypothalamic activities with wild-type ones. As shown in Figure 3F,G, significant reductions in both the frequency and amplitude of the electrical spikes were shown in the DF508 slices as compared to the wild-type ones, suggesting a key involvement of CFTR in electrical firing in the hypothalamus.

### 2.4. CFTR Deficiency Disturbs Mouse Estrus Cycle and Hypothalamic Neuron Activities In Vivo

**Figure 4:**
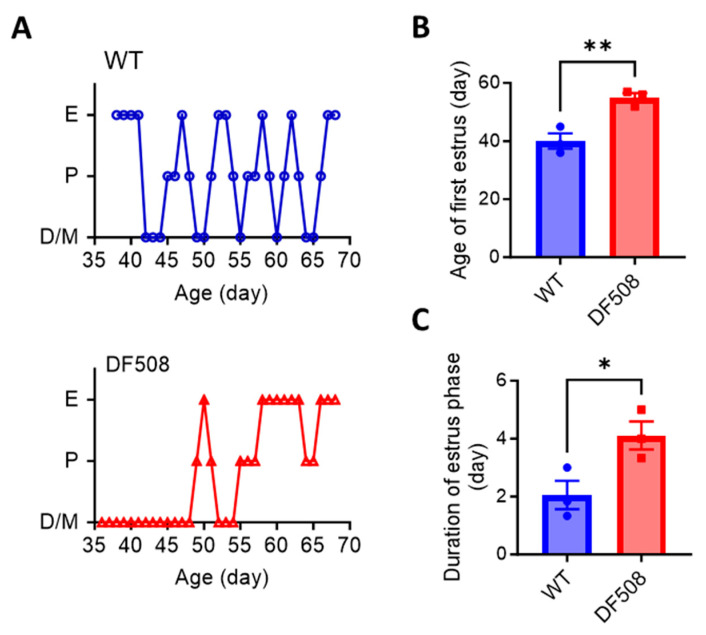
Disturbed estrus cycle and delayed puberty in DF508 mice. (**A**) Recording of estrus cycle including 4 phases, diestrus (D), proestrus (P), estrus (E), and metestrus (M) in WT and DF508 female mice of different ages starting from 35-day-old, close to the onset of puberty in normal mice. (**B**,**C**) Age of first estrus (**B**) and duration of estrus phase (**C**) in WT and DF508 female mice. n = 3. * p < 0.05. ** *p* < 0.01, Two-tail unpaired Student’s *t*-tests. Data are shown as mean ± s.e.m.

**Figure 5:**
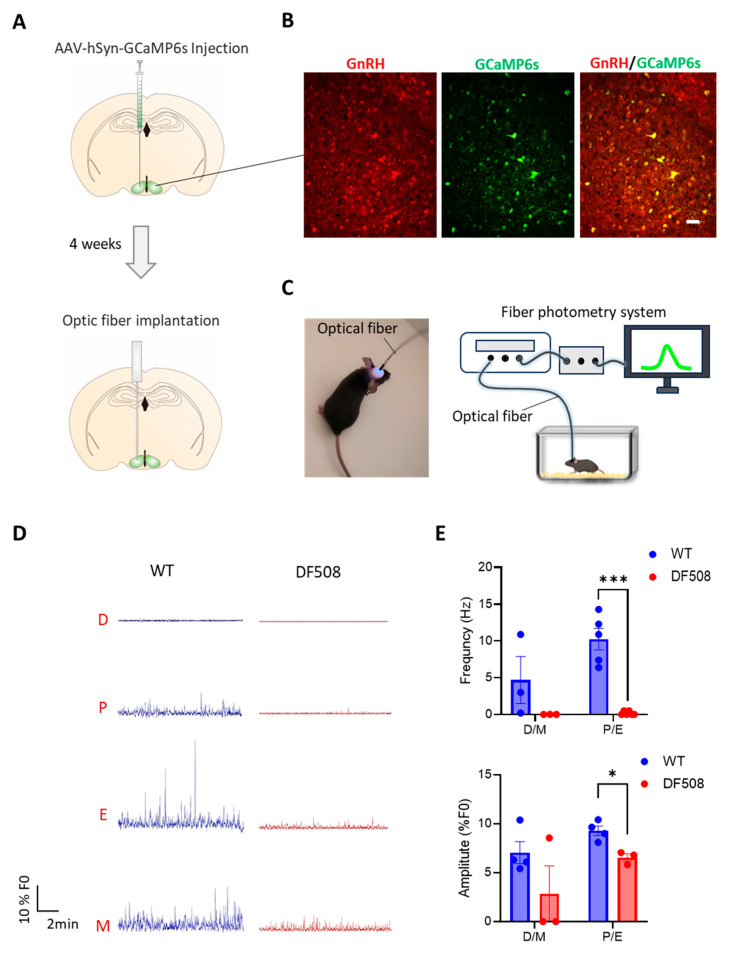
Fiber photometry recording in the hypothalamus of mice. (**A**) Schematic illustration of AAV-hSyn-GCaMP6s virus injection into the mouse hypothalamus brain region followed by optic fiber implantation in 4 weeks. (**B**) Fluorescence image of GCaMP6s (green) and immunofluorescence for GnRH neurons (red) in the hypothalamus tissues of mice after the virus injection. Scale bar, 20 µm. (**C**) Photograph of a mouse after optical fiber implantation (***left***) and schematic diagram (***right***) of GCaMP6s-indicating Ca^2+^ signal recording system with a free-moving mouse in the cage. (**D**) Continuously fiber photometry recording of WT and DF508 female mice at diestrus (D), proestrus (P), estrus (E), and metestrus (M) phases. (**E**) Quantification of Ca^2+^ frequencyand amplitude of WT or DF508 mice in D/M or P/E. * p < 0.05, *** p < 0.001. n = 3–5. Two-tail unpaired Student’s *t*-tests. Data are shown as mean ± s.e.m.

We went on to examine hypothalamic activities in vivo using the DF508 mouse model. Tracking the estrus cycle in the mice over 35 days by vaginal smear (see method) showed that the female DF508 mice exhibited delayed puberty (Figure 4A,B) and a prolonged estrus phase (Figure 4A,C) as compared to the wild-type mice, suggesting estrus cycle disturbance caused by CFTR deficiency. In this model, we performed fiber photometry experiments. Adeno-viruses containing a neuron hSyn promoter and a Ca^2+^ indicator, GCaMP6s, were injected into the hypothalamus (Figure 5A, see method) in the mice. Fluorescence labeling for GnRH in brain tissues from the injected mice showed successful co-expression of GnRH and GCaMP6s in hypothalamic neurons (Figure 5B). Afterward, an optical fiber was surgically implanted into the brain near the hypothalamus in each mouse to monitor GCaMP6s signals in vivo by a fiber-photometry system (Figure 5A,C). Female mice at different estrus cycle phases, namely, diestrus, proestrus, estrus, and metestrus, were used for 20 min continuous detection of Gcamp6-indicating intracellular Ca^2+^ signals at each phase. As shown in Figure 5D, both wild-type and DF508 hypothalamuses were found to be quiescent at the diestrus phase. Starting from the proestrus phase, wild-type mice exhibited hypothalamic Ca^2+^ activities with increasing frequency and amplitude, and the peak signal was detected at the estrus phase. DF508 mice also exhibited some hypothalamic Ca^2+^ activities at the estrus phase, which were however significantly lower than those from the wild-type mice (Figure 5E). These results therefore suggested that estrus-related hypothalamic activities were impaired in DF508 mice.

## 3. Discussion

In summary, using electrophysiology, cell imaging, and fiber photometry approaches on primary cultures, brain slice preparations, or in vivo models, the present study has shown the critical involvement of CFTR in modulating hypothalamic neuron excitability. Though CFTR is shown to mediate either Cl^−^-influx or efflux depending on the cross-membrane Cl^−^ gradient to either suppress or enhance neuronal electrical firing, the loss of CFTR function in the animal model eventually resulted in impairment of hypothalamic neuronal excitability, which may in part account for the disturbed female cycle and reduced female fertility associated with CFTR mutations.

CFTR expression in the nervous systems including hypothalamic neurons has been documented in the past [15,22]. The involvement of CFTR in GnRH production from hypothalamic neurons has also reported [17], although how CFTR contributes to the neuronal functions was not clear. The present study has shown that CFTR-mediated Cl^−^ flow directly modulates hypothalamic neuron electrical activities and Ca^2+^ mobilization, providing mechanisms for its regulatory role in neuronal functions such as GnRH release. Cl^−^ channels are considered either excitatory or inhibitory factors in neurons depending on the electrochemical gradient of Cl^−^ across the membrane [23]. Most studies have claimed CFTR to mediate Cl^−^ efflux depolarizing the membrane [24,25]. Previously, we have also shown in the excitable pancreatic islet β cells that CFTR mediates Cl^−^ efflux, contributing to the firing of action potentials [11]. However, the present study on primary cultures of rat hypothalamic neurons suggested that CFTR could play an inhibitory role under a physiological environmental condition (i.e., ~140 mM extracellular Cl^−^) since the blockage of CFTR facilitated electrical pulses and evoked Ca^2+^ spikes. Given the dependence on extracellular Cl^−^ of such responses to CFTR blockage, CFTR is most likely to mediate Cl^−^ influx into the cells following an inward electrochemical gradient of Cl^−^ in these cells. Notably, the cultured neurons were from the fetus stage of rat brain, which may not be maturely equipped with other Cl^−^ channels/transporters. Therefore, the direction of the electrochemical gradient of Cl^−^ may change over different development stages and dynamic physiological/pathological conditions, which would alter CFTR’s role to be excitatory or inhibitory. According to our data from adult brain slices and adult animals, CFTR should play a positive role in hypothalamic neuron excitability in adults. However, further studies are needed to elucidate whether such a role is directly due to CFTR-mediated Cl^−^ efflux or indirectly by affecting other aspects of the hypothalamic neurons.

The present study showed drastic changes in electrical/Ca^2+^ activities upon extracellular Cl^−^ concentration drop in hypothalamic neurons from either the cultures or brain slides, which indicates the importance of Cl^−^ environment and Cl^−^ transport in maintaining the neuronal functions [26]. It should be noted that in the hypothalamic neuron cultures, the reduction of Cl^−^ elevated the Ca^2+^ level, while in brain slices, Cl^−^ reduction suppressed electrical pulses, which are seemingly contradicting. A possible explanation is that the sustained Ca^2+^ elevation in the neurons caused by Cl^−^ reduction may be detrimental to the neurons affecting the generation of new action potentials as previously reported [27]. Another explanation would be the intrinsic difference between isolated/cultured neurons and neurons with relatively intact cell-cell network in the brain slices. Non-neuron cells or non-hypothalamic neurons in the brain slices could be affected by the low Cl^−^ environment to indirectly suppress the hypothalamic neurons in the brain slices. Of note, in Ca^2+^ imaging experiments, we were able to differentiate neurons and glia cells, which revealed glia cells to be much less sensitive to Cl^−^ reduction or CFTR inhibition than the neurons. Therefore, the effect of Cl^−^ removal or CFTR deficiency in impairing the hypothalamic neuron activities observed in brain slices or in intact animals presently may primarily be the effects on neurons. Further studies could be conducted to understand Cl^−^ environment and CFTR in neuronal network and neuron-glia interactions.

Importantly, the present study has used a recently developed technology, Gcam6s-fiber-photometry [28,29], and shown in vivo hypothalamic electrical/Ca^2+^ activities in female mice over different periods of the estrus cycle. Correlating these fiber-photometry data with the estrus cycle phases suggested enhanced hypothalamic activities in proestrus/estrus phase, which is in line with the documented increase in GnRH release in this phase compared to the diestrus phase [30,31]. The impaired hypothalamic activity seen in DF508 at estrus may account for dysregulated GnRH release and disturbed estrus cycle with CFTR deficiency [32]. It should be noted that CFTR has been reported to affect female reproduction in multiple aspects [33,34,35,36]. Ovarian hormone production is affected by CFTR deficiency in ovarian granulosa cells too [37]. The low hypothalamic activity observed in DF508 mice may also be a result of ovarian dysfunction. Nevertheless, the present study combining in vitro and in vivo approaches has provided direct evidence to suggest the role of CFTR in modulating hypothalamic neuron excitability. Similar approaches could be used to further understand CFTR in other types of neurons or other excitable cell types.

## 4. Methods and Materials

### 4.1. Animals

Mice or rats were purchased from Centralized Animal Facilities (CAF) at Hong Kong Polytechnic University or the Laboratory Animal Services Centre at The Chinese University of Hong Kong. All animals were maintained at CAF and experimentally handled with procedures approved by the Animal Subjects Ethics Sub-committee at Hong Kong Polytechnic University (19-20/27-BME-R-OTHERS, 20-21/259-BME-R-HMRF).

### 4.2. Primary Cultures of Hypothalamic Neurons

Primary hypothalamic neurons were collected from rat fetuses on embryonic day 19. The hypothalamus tissues were isolated from the brains in ice-cold neurobasal medium (21103049, Thermo Fisher Scientific, Waltham, MA, USA), digested in 0.25% trypsin at 37 °C for 15 min. The cells were washed with neurobasal medium containing 10% fetal bovine serum (FBS), 0.25% L-glutamine, 1% penicillin-streptomycin, and 0.1% dNase, pipetted gently and centrifuged at 1000 rpm for 5 min at room temperature. The supernatant was discarded, and the cells were resuspended in the abovementioned medium, plated at 1 × 10^5^ cells in 35 mm culture or confocal dishes coated with 50 μg/mL poly-lysine (P8920, Sigma-Aldrich, St. Louis, MO, USA). The medium was changed to a neurobasal medium containing 2% B27, 0.25% L-glutamine, and 1% penicillin-streptomycin. The medium was half-changed every 72 h.

### 4.3. Patch-Clamp

Primary hypothalamic neurons were cultured on 35 mm culture dishes for 10 days before patch-clamp recording. Borosilicate glass-made patch pipettes were pulled with a micropipette puller (P-1000, Sutter Instrument, Novato, CA, USA) to a resistance of 5–7 MΩ before filled with a pipette solution (in mM): KCl 138, NaCl 10, MgCl_2_ 1, Glucose 10 and HEPES 10 (pH 7.4). Membrane potentials of cells were recorded with a patch-clamp amplifier (Axon Instruments Multiclamp700B, Molecular Devices, San Jose, CA, USA) and a data acquisition system (Axon Instruments DigiData1550B, Molecular Devices, San Jose, CA, USA). Cells were bathed in a solution containing (in mM): NaCl 130, KCl 5, MgCl_2_ 1, CaCl_2_ 2.5, Glucose 10, HEPES 20 (pH 7.4). When the whole-cell giga seal was formed, the membrane potential of cells was measured by current clamp step recording (the injected currents from 0 to 500 pA with 50 pA increment, 0.8 ms). The cells with action potentials evoked by injected currents were considered hypothalamic neurons.

### 4.4. Ca^2+^ Imaging

Cells seeded on the glass-bottom dishes (150680, Thermo Fisher Scientific, Waltham, MA, USA) were washed with the bath solution as used in patch-clamp, incubated with Fura-2 (3 µM) (F1200, Thermo Fisher Scientific, Waltham, MA, USA) in the bath solution at 37 °C for 30 min, then mounted on to a fluorescence microscope (Eclipse Ti, Nikon, Tokyo, Japan) for intracellular Ca^2+^ measurement. Fluorescence was alternatively excited by dual wavelength 340 and 380 nm with an interval of 3 s, and the emitted fluorescent lights were collected at 510 nm. Some experiments used a low Cl^−^ solution containing (in mM): sodium D-gluconate 128, NaCl 2, KCl 5, MgCl_2_ 1, CaCl_2_ 2.5, Glucose 10, HEPES 20 (pH 7.4).

### 4.5. Brain Slice Patch-Clamp

C57 mice around 10 weeks old were used. The brain-slice-preparing procedures were conducted as previously reported [38]. Briefly, the mouse was anesthetized with ketamine (100 mg/kg) and xylazine (10 mg/kg) followed by heart perfusion with an NMDG-HEPES-based solution (in mM): NMDG-Cl 92, KCl 2.5, NaH_2_PO_4_ 1.25, NaHCO_3_ 30, HEPES 20, Glucose 25, Thiourea 2, Na-ascorbate 5, Na-pyruvate 3, CaCl_2_ 0.5, and MgSO_4_ 10, pH 7.3–7.4. The mouse was then decapitated and the brain was rapidly isolated and submerged in ice-cold (4 °C) and oxygenated NMDG-HEPES solution. Coronal slices (300 µm) containing the hypothalamus were cut with a vibratome (VT1000S, Leica, Wetzlar, Germany) in ice-cold NMDG-HEPES solution. The slices were incubated at NMDG-HEPES solution at 35 °C for 10 min, switched to oxygenated Na^+^ based solution (in mM): NaCl 92, KCl 2.5, NaH_2_PO_4_ 1.25, NaHCO_3_ 30, HEPES 20, Glucose 25, Thiourea 2, Na-ascorbate 5, Na-pyruvate 3, CaCl_2_ 2, and MgSO_4_ 2, pH 7.3–7.4 for 1 h incubation before patch-clamp recording. Brain slice was placed on the hold chamber and perfused with an oxygenated solution (in mM): NaCl 120, MgCl_2_ 2, CaCl_2_ 2, KCl 3, NaH_2_PO_4_ 1.2, NaHCO_3_ 23, Glucose 11, pH 7.3–7.4. The brain-slice patch-clamp technique was conducted using a patch-clamp system (DigiData 1322A, HEKA Instruments, Southboro, MA, USA). For low Cl^−^ treatment, a low Cl^−^ solution (in mM): sodium D-gluconate 120, MgSO_4_ 2, calcium gluconate 2, KCl 3, NaH_2_PO_4_ 1.2, NaHCO_3_ 23, Glucose 11, pH 7.3–7.4 was perfused.

### 4.6. Vaginal Cytology

The estrous cycle was assessed using the vaginal cytology method [39]. Vaginal smears were performed daily for mice starting from 35 days old for a consecutive 50 days. Collected vaginal cells were stained with hematoxylin (H3136, Sigma-Aldrich, St. Louis, MO, USA) for 3 min followed by eosin (861006, Sigma-Aldrich, St. Louis, MO, USA) for 3 min. The identification of the estrous cycle stage was based on the presence or absence of leukocytes, cornified epithelial cells, and nucleated epithelial cells, as described by Felicio et al. [40].

### 4.7. Stereotaxic Surgery

The mouse was anesthetized by ketamine (100 mg/kg) and xylazine (10 mg/kg) and positioned in the stereotaxic apparatus. The skin above the skull was clipped and the ointment was applied on the eyes. Then, 1.0 μL of virus (pAAV-hSyn-GCaMP6s, OBiO Technology, Shanghai, China) was delivered to the hypothalamus brain region (AP, −1.2 mm, ML, −0.3 mm, DV, 6.0 mm) at 0.1 μL/min by a microinjection system (Nanoliter 2010, World Precision Instruments, Sarasota, FL, USA) through a craniotomy (<1 mm^2^). After the virus injection, the needle for virus injection was retracted slowly. The puncture site in the skulls was disinfected and sutured. 4 weeks later, the optic fiber was implanted (AP, −1.2 mm, ML, −0.3 mm, DV, 6.0 mm) and fixed to the skull with dental cement. The skin around the dental cement was disinfected.

### 4.8. Fiber Photometry

After fiber implantation, mice were anesthetized with isoflurane (1.0 to 2.5% in O_2_) before the fiber photometry system (Thinker Tech Nanjing BioScience, Nanjing, China) was wired with the fiber on each mouse. After the mouse woke up in the open field, GCaMP6s fluorescence was captured using the fiber photometry system. The excitation and receiving wavelengths for fiber photometry were 470 nm with 30 nm bandwidth and 510 nm with 25 nm bandwidth, respectively. The GCaMP6s fluorescence signals (F) were collected at 100 Hz and analyzed using a customized MATLAB script. “F0” was the baseline of the fluorescence signal. The GCaMP6s fluorescence changes ΔF, calculated as (F − F0)/F0, equal to or higher than 0.05 were included in the frequency and amplitude calculation. Each mouse was recorded for 20 min.

### 4.9. Immunofluorescence Staining

Brain tissues were harvested after whole-body perfusion by 4% paraformaldehyde (PFA) and fixed by immersion PFA overnight at 4 °C. After being washed three times in PBS, brain tissues were cryoprotected in 30% sucrose in PBS at 4 °C for 24 h and mounted in OCT embedding media (Tissue-Tek 4583, Sakura Finetek, Torrance, CA, USA). Starting from the injection site, around 10 continuous coronal brain slices with an interval of 10 μm were collected on adhesion microscope slices. Slices were blocked with 10% goat serum, 5% BSA, and 0.3% Triton X-100 in PBS at room temperature for 1 h and incubated with primary antibodies (GnRH, 1:500, ab281844, Abcam, Cambridge, UK) overnight at 4 °C, and subsequently fluorochrome-conjugated secondary antibody (goat anti-rabbit IgG (H+L) Alexa Fluor 594, 1:1000, A-11012, Thermo Fisher Scientific, Waltham, MA, USA) for 1 h at room temperature. Images were acquired with a fluorescence microscope (Nikon Eclipse Ti2, Tokyo, Japan).

### 4.10. Statistics

Data are represented as mean ± s.e.m. Two-tail unpaired Student’s *t* test was used for two group comparisons. Two-way ANOVA was used when there were two different categorical independent variables. *p* values below 0.05 were considered significant. All graphs were generated, and all statistical analyses were conducted with GraphPad Prism 9.
